# Cdk1 Phosphorylates *Drosophila* Sas-4 to Recruit Polo to Daughter Centrioles and Convert Them to Centrosomes

**DOI:** 10.1016/j.devcel.2016.05.022

**Published:** 2016-06-20

**Authors:** Zsofia A. Novak, Alan Wainman, Lisa Gartenmann, Jordan W. Raff

**Affiliations:** 1 Sir William Dunn School of Pathology, University of Oxford, South Parks Road, Oxford OX1 3RE, UK

## Abstract

Centrosomes and cilia are organized by a centriole pair comprising an older mother and a younger daughter. Centriole numbers are tightly regulated, and daughter centrioles (which assemble in S phase) cannot themselves duplicate or organize centrosomes until they have passed through mitosis. It is unclear how this mitotic “centriole conversion” is regulated, but it requires Plk1/Polo kinase. Here we show that in flies, Cdk1 phosphorylates the conserved centriole protein Sas-4 during mitosis. This creates a Polo-docking site that helps recruit Polo to daughter centrioles and is required for the subsequent recruitment of Asterless (Asl), a protein essential for centriole duplication and mitotic centrosome assembly. Point mutations in Sas-4 that prevent Cdk1 phosphorylation or Polo docking do not block centriole disengagement during mitosis, but block efficient centriole conversion and lead to embryonic lethality. These observations can explain why daughter centrioles have to pass through mitosis before they can duplicate and organize a centrosome.

## Highlights

• Cdk1 phosphorylates Sas-4 to initiate Polo/Plk1 recruitment to daughter centrioles
• Polo recruitment promotes Asterless (Asl) incorporation into daughter centrioles
• Asl incorporation licenses new centrioles to duplicate and organize centrosomes
• These observations help explain why centriole conversion is tied to mitosis

## Graphical Abstract

**Figure:**
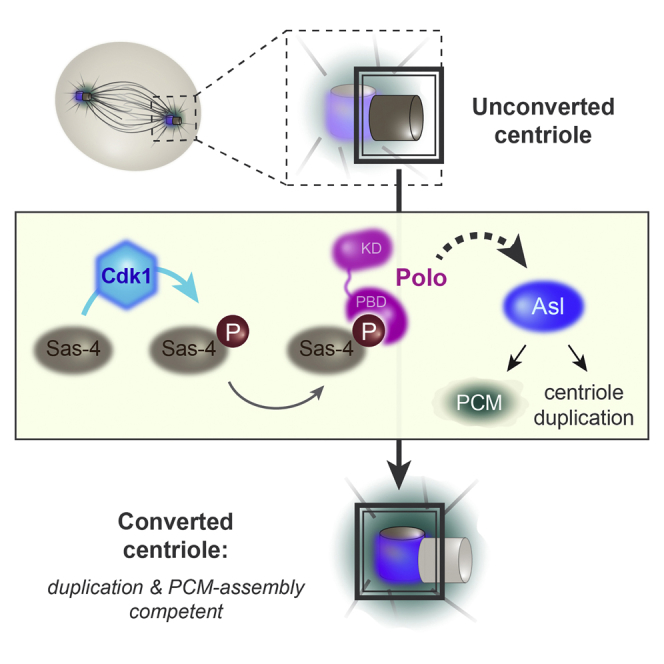

## Teaser

Newly formed centrioles are converted to duplication-competent centrosomes during their first mitosis. Novak et al. show that in *Drosophila* this centriole conversion is induced by the Cdk1-dependent phosphorylation of Sas-4. Phosphorylated Sas-4 recruits Polo/Plk1 to new centrioles and promotes Asl incorporation, allowing these centrioles to duplicate and assemble a centrosome.

## Introduction

Centrioles organize centrosomes and cilia. These organelles have many important functions in cells, and their dysfunction has been linked to several human diseases (Bettencourt-Dias et al., 2011, Conduit et al., 2015a, Nigg and Raff, 2009). Much attention has focused on the mechanisms that regulate centriole (and so centrosome and cilium) numbers, as numerical abnormalities can be highly deleterious to cells. Centriole loss gradually triggers a p53-dependent response that leads to cell-cycle arrest and/or cell death in mammalian cells (Izquierdo et al., 2014, Lambrus et al., 2015, Wong et al., 2015), while centriole amplification has been linked to cancer (Basto et al., 2008, Ganem et al., 2009, Godinho et al., 2014, Gönczy, 2015) and more recently to microcephaly (Arquint and Nigg, 2014, Marthiens et al., 2013). How centriole amplification influences these pathologies remains unclear (Serçin et al., 2016, Vitre et al., 2015).

Most cells are born with two centrioles that are precisely duplicated in S phase when a new “daughter” centriole is assembled at right angles to each “mother” in a tightly apposed engaged configuration. This configuration appears to be crucial for regulating centriole numbers, as mother centrioles normally cannot duplicate again until they disengage from their daughters during the subsequent mitosis, a process that requires the mitotic kinase Polo/Plk1 (Loncarek et al., 2010, Shukla et al., 2015, Tsou and Stearns, 2006a, Tsou et al., 2009). Thus, mother centrioles normally only duplicate once in S phase, and disengagement functions as a “license” that is acquired during mitosis to enable mother centrioles to duplicate again during the next S phase (Fırat-Karalar and Stearns, 2014, Nigg, 2007, Tsou and Stearns, 2006b).

Another important mechanism that helps regulate centriole numbers is that only mother centrioles are competent to duplicate during S phase (Cunha-Ferreira et al., 2009, Kleylein-Sohn et al., 2007, Loncarek et al., 2008). The restriction that prevents newly assembled daughter centrioles from duplicating during S phase does not simply rely on them being engaged to their mothers: under certain experimental conditions, such as overexpression of Sak/Plk4 (the key protein kinase that initiates centriole duplication), mother centrioles can duplicate again and form multiple daughters, but daughters cannot form daughters of their own. Importantly, it was recently shown that newborn daughter centrioles cannot form centrosomes or duplicate until they have passed through mitosis and are modified by Polo/Plk1 (Wang et al., 2011); this process has been termed “centriole-to-centrosome” conversion (Fu et al., 2016, Izquierdo et al., 2014, Wang et al., 2011), although we sometimes use the more generic “mitotic centriole conversion” here (see Discussion).

Great progress has been made in understanding the molecular mechanisms of centriole assembly (Conduit et al., 2015a, Gönczy, 2012, Jana et al., 2014), and recent studies have shed some light on the process of mitotic centriole conversion. In human cells, Cep295 is required for this process: in the absence of Cep295, new daughter centrioles disengage from their mothers during mitosis but they cannot recruit any pericentriolar material (PCM) and these centrioles are destabilized (Izquierdo et al., 2014). In flies, the conserved centriole protein Asterless (Asl) is essential for both centriole duplication and for mitotic PCM recruitment (Blachon et al., 2008, Bonaccorsi et al., 1998, Dobbelaere et al., 2008, Varmark et al., 2007). Asl helps recruit Sak/Plk4 to the mother centriole to initiate centriole duplication (Dzhindzhev et al., 2010, Novak et al., 2014), and to recruit Spd-2 and Cnn to the mother centriole to initiate mitotic centrosome assembly (Conduit et al., 2014a, Conduit et al., 2014b). It was recently shown that Asl is only recruited to daughter centrioles during mitosis, at about the time that they are converted to centrioles that can recruit PCM and duplicate (Fu et al., 2016, Novak et al., 2014). Thus, the recruitment of Asl to newly disengaged centrioles during mitosis appears to be a crucial step in centriole conversion in flies. Interestingly, a recent study showed that the fly homolog of Cep295, Ana1, also plays an important part in centriole conversion in flies (Fu et al., 2016). Ana1 is recruited to centrioles by Cep135/Bld10 in interphase, and is subsequently required to recruit Asl to the newborn centrioles during mitosis; a similar mechanism appears to operate in human cells (Fu et al., 2016). These previous studies, however, provide no insight into the crucial question of how Asl recruitment is regulated during the cell cycle so that it only occurs during mitosis.

We previously showed that another conserved centriole protein, Sas-4, has an important role in initially recruiting Asl to newborn centrioles during mitosis in flies (Novak et al., 2014), and a direct interaction between Sas-4 and Asl (human CPAP and Cep152, respectively) has been observed in both flies and humans (Cizmecioglu et al., 2010, Dzhindzhev et al., 2010, Hatch et al., 2010). Unlike Asl, Sas-4 is recruited to daughter centrioles during their assembly in S phase (Conduit et al., 2015b, Novak et al., 2014). If Sas-4 helps recruit Asl to newborn centrioles, this function must be strictly regulated so that it only occurs during mitosis. In this study we set out to identify the mechanism that might regulate the ability of Sas-4 to promote the recruitment of Asl to daughter centrioles during mitosis. We find that Sas-4 is phosphorylated during mitosis on Thr200 by the master mitotic regulator Cdk1 to create a Polo-docking site that appears to be required to recruit Polo, and subsequently Asl, to new centrioles. Remarkably, single point mutations that perturb Sas-4-Thr200 phosphorylation or the creation of the Polo-docking site strongly perturb Polo and Asl recruitment to the new centriole during mitosis. Although these newborn centrioles can separate from their mothers during mitosis, they are not converted to mother centrioles that can duplicate or recruit PCM, and embryos expressing these mutant Sas-4 proteins die very early in development.

## Results

### Sas-4 Thr200 Is Required to Recruit Asl to New Centrioles

To investigate whether the ability of Sas-4 to recruit Asl to centrioles might be regulated by cell-cycle-dependent phosphorylation we searched for regions within, or adjacent to, the Asl-interacting region of Sas-4 (amino acids [aa] 101–650) (Dzhindzhev et al., 2010) that contained consensus phosphorylation sites for either Cyclin-dependent kinases (Cdks) or Polo/Plk kinases and that were conserved in *Drosophila* species. We identified four regions for further analysis: I (aa 198–244), II (aa 257–304), III (aa 378–406), and IV (aa 654–686) (Figure S1A).

**Figure 1:**
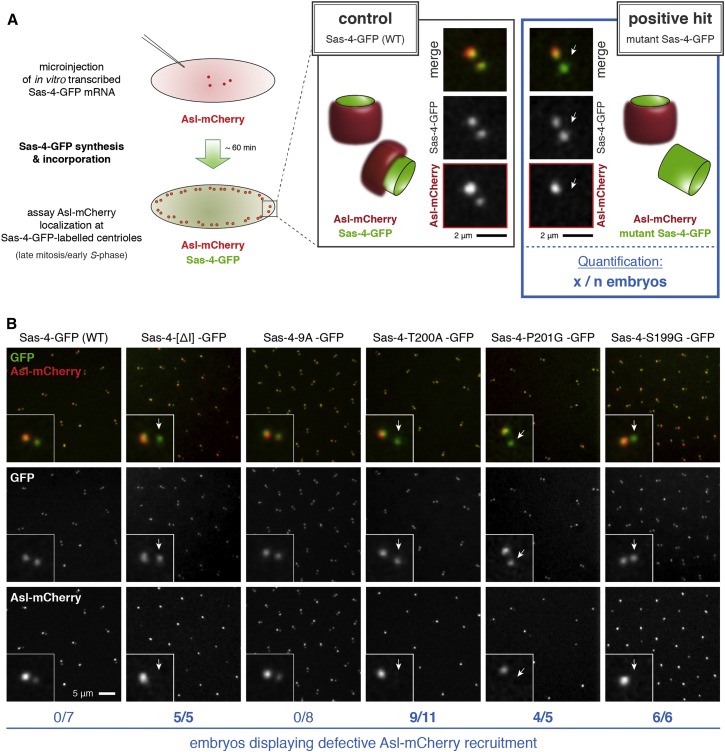
Sas-4-Thr200 Is Required to Localize Asl to New Centrioles (A) Schematic and example images illustrate the RNA injection assay used for screening the role of various mutant Sas-4-GFP proteins (green) in the recruitment of Asl-mCherry (red) to new centrioles. Arrows in micrographs highlight a positive hit, where Asl-mCherry has not detectably incorporated into the new centriole that has just separated from its older mother. Scale bars, 2 μm. (B) Asl-mCherry (red) localization at newly separated centrioles in living embryos expressing various Sas-4-GFP fusions from injected mRNA (green, as indicated). Note that Asl-mCherry localizes normally at new centrioles in embryos expressing WT Sas-4-GFP or Sas-4-9A-GFP, in which nine conserved Ser/Thr residues in the vicinity of Thr200 were substituted to Ala (see Figure S2B). The localization of Asl-mCherry is disrupted in embryos expressing Sas-4-[ΔI]-GFP or embryos expressing full-length Sas-4-GFP carrying either T200A, P201G, or S199G point mutations. Arrows indicate new centrioles that have not incorporated Asl-mCherry. Scale bar, 5 μm. See also Figures S1 and S2.

We synthesized mRNA in vitro encoding GFP fusions of either wild-type (WT) Sas-4 (Sas-4-GFP) or mutant versions in which these regions were individually deleted. We injected these mRNAs into fly embryos expressing Asl-mCherry; the Sas-4-GFP proteins are gradually synthesized from the injected mRNA and incorporated into centrioles in competition with endogenous, unlabeled, Sas-4. We then assayed the effect of each fusion protein on Asl-mCherry incorporation (see Figure 1A for a schematic summary of this assay). In all cases, the Sas-4-GFP signal appeared as a tight, symmetrical focus at newly separated centriole pairs, demonstrating that none of the deletions blocked Sas-4 targeting to centrioles (Figures 1B and S1B). Asl normally localizes asymmetrically to newly separated centriole pairs because it only starts to be incorporated into daughter centrioles during mitosis, so new mother centrioles contain very little Asl (Novak et al., 2014), and Asl-mCherry behaved in this way in all embryos expressing Sas-4-GFP, Sas-4-[ΔII]-GFP; Sas-4-[ΔIII]-GFP, or Sas-4-[ΔIV]-GFP (Figures 1B and S1B). In contrast, the recruitment of Asl-mCherry to new centrosomes at the end of mitosis was greatly reduced in embryos expressing Sas-4-[ΔI]-GFP (note that Asl-mCherry localization in all these experiments was scored blindly) (Figures 1B and S1B).

Although Sas-4-[ΔI]-GFP expression severely disrupted the recruitment of Asl-mCherry to newly disengaged daughter centrioles, it did not detectably disrupt the localization of Asl-mCherry at newly disengaged mother centrioles (Figures 1B and S1B). This is expected, as fractions of both Sas-4 and Asl are stably incorporated into centrioles (Conduit et al., 2015b, Novak et al., 2014) and Asl-mCherry was initially recruited to these older centrioles during earlier rounds of mitosis: as Sas-4[ΔI]-GFP is being gradually translated in these experiments, the ratio of Sas-4[ΔI]-GFP/endogenous-Sas-4 at centrioles gradually increases during successive rounds of centriole duplication, presumably until it reaches a critical level that is sufficient to perturb Asl-mCherry recruitment to the new daughter centrioles (see Figure 1A).

Region I of Sas-4 is required to promote Asl recruitment to newly disengaged daughter centrioles and contains several Ser/Thr residues that are highly conserved in *Drosophila* species (Figure S2A). We tested whether mutating any of these residues to Ala would lead to a defect in Asl-mCherry recruitment (Figure S2). The individual expression of several forms of Sas-4-GFP containing combinations of multiple Ser/Thr-to-Ala substitutions within region I, including a form in which nine Ser/Thr residues were mutated to Ala (Sas-4-9A), did not detectably perturb Asl-mCherry recruitment (Figures 1B, S2B, and S2C; and data not shown). In contrast, expressing a form of Sas-4-GFP in which the single residue Thr200 was mutated to Ala (Sas-4-T200A-GFP) gave a strong defect in Asl-mCherry recruitment (Figures 1B and S2C). We conclude that Sas-4 Thr200 is required to promote Asl recruitment.

### Sas-4 Thr200 Can Be Phosphorylated by Cdk1 to Create a Polo-Docking Site In Vitro

**Figure 2:**
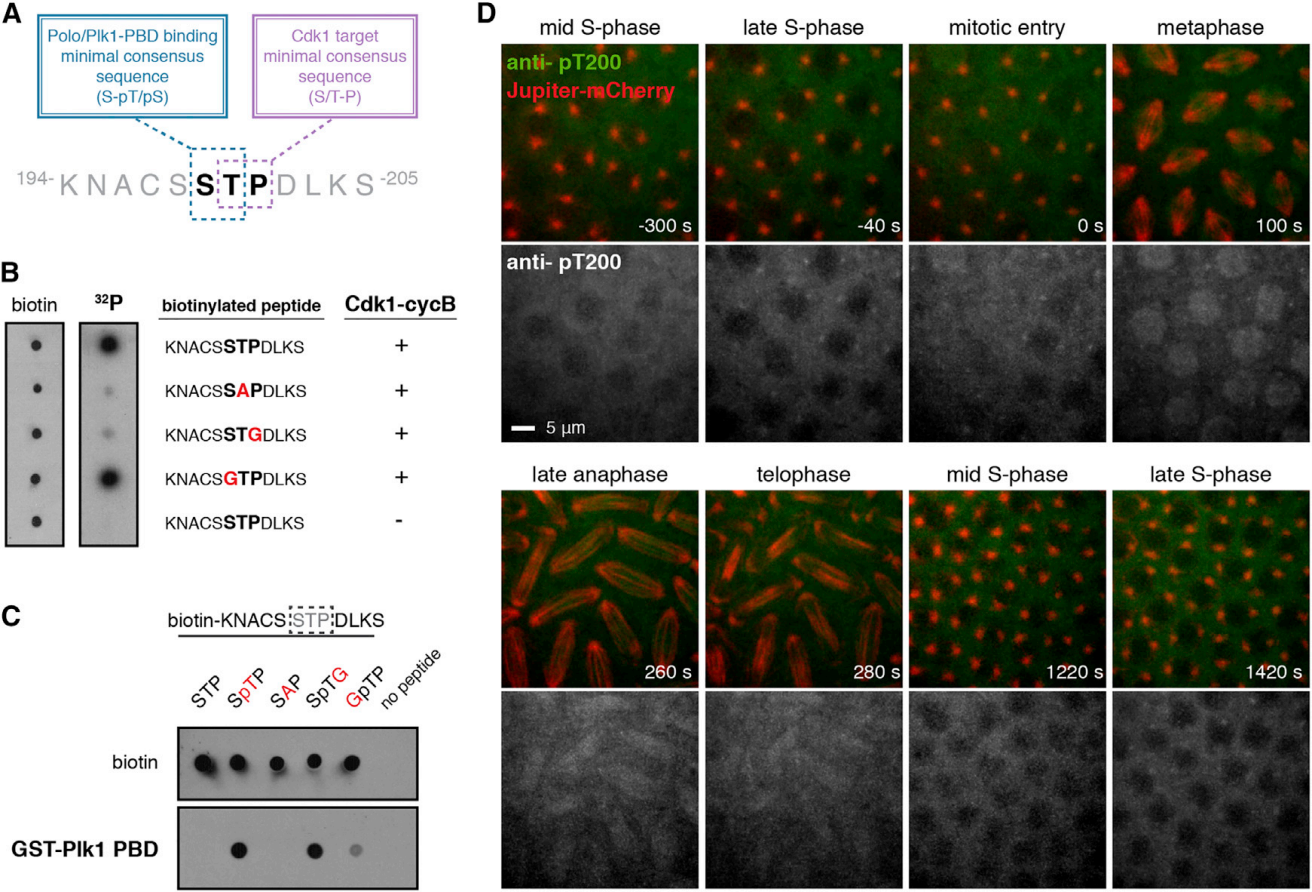
Sas-4-Thr200 Is Phosphorylated by Cdk1 to Create a Polo-Docking Site In Vitro and Appears to be Phosphorylated from Late S Phase to Early Mitosis In Vivo (A) Amino acid sequence of the *Drosophila* Sas-4-Thr200-STP motif. The key residues required for Cdk1-dependent phosphorylation and binding of the Polo/Plk1 Polo-Box are indicated. (B) In vitro assay of Cdk1/Cyclin B-dependent phosphorylation of the *Drosophila* Sas-4-Thr200-STP motif. Peptide sequences around the STP motif and the presence, or absence, of Cdk1/Cyclin B are indicated. The dot blot shows the loading of the biotinylated peptides (left panel); the autoradiogram shows the incorporation of ^32^P (right panel). (C) In vitro assay of the *Drosophila* Sas-4-Thr200-STP motif binding to recombinant GST-Polo-Box protein. Peptide sequences are indicated. The dot blots show the loading of peptide (top panel), and the binding of GST-Polo-Box domain (bottom panel). (D) Anti-Sas-4-pThr200 antibodies (green in merged panels, white in grayscale panels; note the antibody is difficult to visualize at centrosomes in the merged panels) were injected into embryos expressing Jupiter-mCherry (red). Time (s) relative to mitotic entry (nuclear envelope breakdown; t = 0 s) is indicated. Note that the antibodies do not detectably bind centrosomes during mid-S phase (t = −300 s) but start to accumulate at centrosomes during late S phase (t = −40 s) shortly before nuclear envelope breakdown. During anaphase the antibodies are gradually lost from the spindle poles, but they reappear at centrosomes shortly before mitotic entry during the subsequent cell cycle. Ten of ten injected embryos showed the same temporal pattern of antibody localization at centrosomes as in this example. Scale bar, 5 μm. See also Figure S3.

In flies, Sas-4 Thr200 is followed by a conserved proline residue (Pro201; Figure 2A), suggesting that it might be a substrate for Cdks (Endicott et al., 1999); it is also preceded by a conserved serine residue (Ser199; Figure 2A), suggesting that Thr200, when phosphorylated, could act as a docking site for Polo kinase (Plk1 in humans), which can interact with phosphorylated SpS/pT motifs through its conserved Polo-box domain (PBD) (Lowery et al., 2005). A peptide containing the fly Sas-4-Thr200-STP motif was efficiently phosphorylated by recombinant human Cdk1/Cyclin B in vitro, and the phosphorylation was dramatically reduced if either Thr200 or Pro201 (but not Ser199) was mutated (Figure 2B). The peptide also functioned as an efficient docking site for a recombinant human GST-PBD fusion protein in vitro, but only when Thr200 was phosphorylated; binding was also dramatically reduced when Ser199 was mutated (Figure 2C). We conclude that the Sas-4-Thr200-STP motif can be phosphorylated by Cdk1/Cyclin B to create a Polo-docking site in vitro. Interestingly, although the Asl/Cep152-interacting region of Sas-4/CPAP is generally not well conserved between flies and vertebrates, vertebrate CPAP proteins also contain a highly conserved STP motif in this region—surrounding human CPAP Thr616—and this motif can also be phosphorylated by Cdk1/Cyclin B to create a Plk1-docking site in vitro (Figures S3A–S3C).

### The Sas-4-Thr200-STP Motif Is Required to Recruit Asl to Centrioles

To test whether the recruitment of Asl to new centrioles required Cdk1 phosphorylation (dependent on Pro201, in vitro) and/or the creation of the Polo-docking site (dependent on Ser199, in vitro), we expressed several forms of Sas-4-GFP in which either Pro201 or Ser199 were mutated. Both Sas-4-P201G-GFP and Sas-4-S199G-GFP produced a strong defect in Asl-mCherry recruitment (Figure 1B), supporting the hypothesis that Asl recruitment is dependent on both Cdk1 phosphorylation and the creation of a Polo-docking site. Moreover, the conservative substitution of Ser199 to Thr (Sas-4-S199T) also strongly disrupted Asl-mCherry recruitment (Figure S2C), suggesting that the crucial function of Ser199 is not to be phosphorylated, but rather to recruit the PBD. Finally, we also tested the effect of substituting Thr200 for a “phospho-mimicking” Glu. The negative charge of Glu can mimic the negative charge of the phosphate group, potentially allowing Glu to engage in electrostatic interactions in a similar manner to phospho-Thr. Importantly, however, the interaction between a phospho-peptide and the PBD is only partially mediated by electrostatic interactions, as it also relies on specific hydrogen-bonding interactions between the phosphate group and the PBD (Elia et al., 2003), so PBD binding to phospho-Thr should not be mimicked by Glu. The expression of Sas-4-T200E-GFP strongly disrupted Asl-mCherry recruitment (Figure S2C), demonstrating that Glu cannot mimic the function of phospho-Thr in Asl recruitment. Taken together, these studies support the hypothesis that the Sas-4-Thr200-STP motif functions as a Cdk1-dependent Polo-docking site that is required to recruit Asl to newborn centrioles during mitosis in vivo.

### Sas-4-Thr200 Appears to be Phosphorylated during Mitosis In Vivo

To test whether Thr200 is phosphorylated in vivo, we generated antibodies against a phospho-Thr200-containing peptide and purified antibodies that recognized the phosphorylated, but not the non-phosphorylated, peptide (Figure S3D). In fixed embryos these antibodies did not detectably stain centrosomes (not shown), perhaps because phosphorylated Thr200 rapidly binds Polo, which then masks the phosphorylated epitope. In an attempt to overcome this potential problem, we fluorescently labeled the antibodies and injected them into living embryos that expressed the microtubule (MT) marker Jupiter-mCherry to allow the accurate staging of cell-cycle progression (Figure 2D).

We previously showed that fluorescently labeled antibodies raised against non-phosphorylated Sas-4 bind to centrosomes throughout the cell cycle when injected into embryos (Novak et al., 2014). In contrast, the anti-Sas-4-pThr200 antibodies did not detectably bind to centrosomes in early S phase (Figure 2D, t = −300 s) but started to accumulate there in late S phase, reaching maximal levels (Figure 2D, t = −40 s) just before nuclear envelope breakdown (Figure 2D, t = 0 s). Antibody levels at centrosomes remained high until metaphase (Figure 2D, t = 100 s) and then fell during anaphase (Figure 2D, t = 260 s), and the antibody was essentially undetectable at centrosomes by telophase (Figure 2D, t = 280 s). Importantly, the antibodies started to bind to centrosomes again toward the end of the next S phase (Figure 2D, t = 1,420 s), demonstrating that they were not simply degraded or inactivated in the embryo. Thus, Sas-4 Thr200 appears to be phosphorylated in vivo from late S phase to late mitosis. This strongly suggests that Sas-4-Thr200 phosphorylation is catalyzed by the M-phase-specific Cdk1, rather than by the S-phase-specific Cdk2.

### The Sas-4-Thr200-STP Motif Is Required to Recruit Polo to New Centrioles but Is Dispensable for Centriole Disengagement

**Figure 3:**
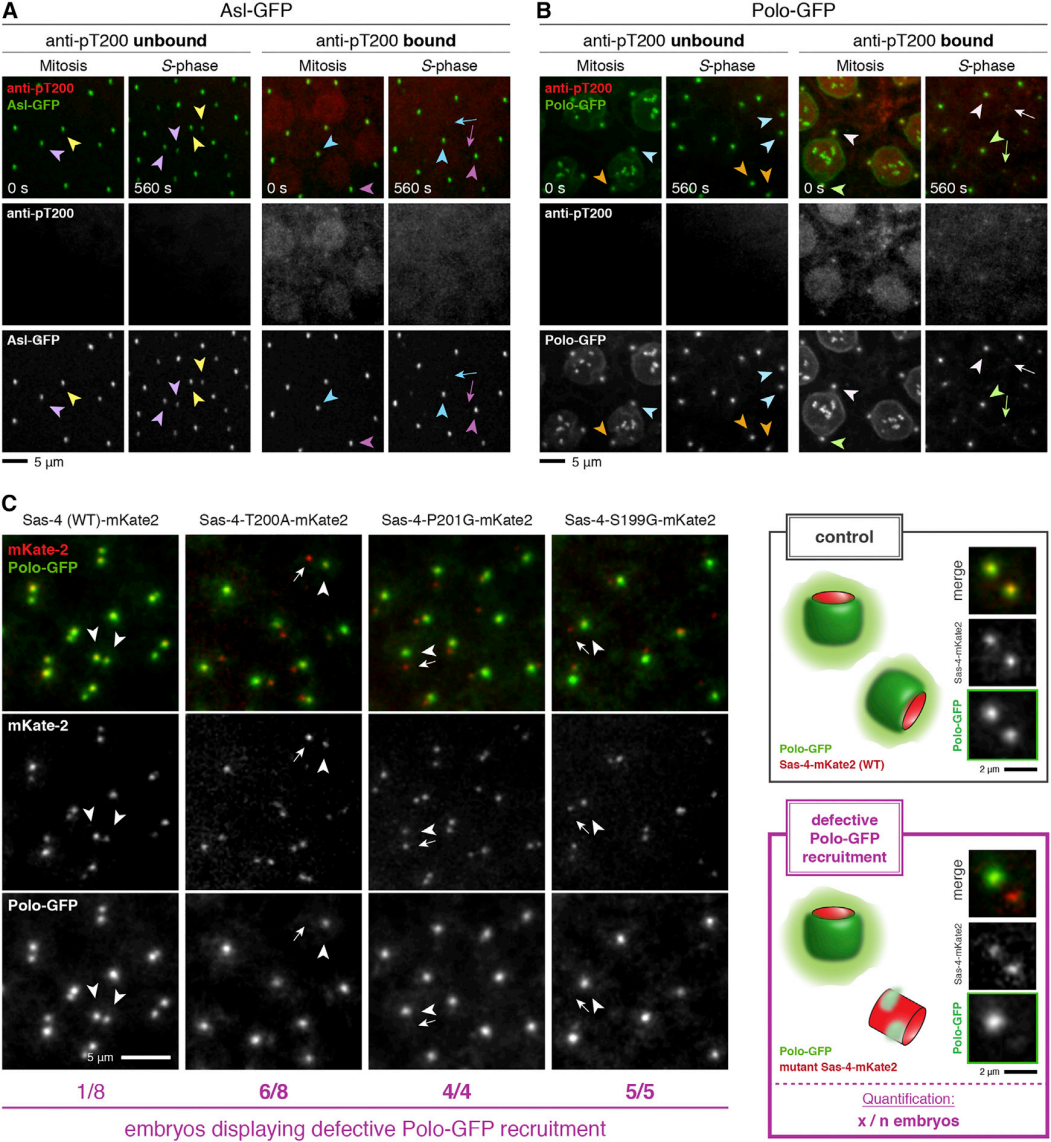
The Sas-4-Thr200-STP Motif Helps Recruit Both Polo and Asl to New Centrioles (A and B) Asl-GFP (A, green) and Polo-GFP (B, green) localization in embryos injected with high levels of anti-Sas-4-pThr200 antibodies (red). Embryos are shown during mitosis and the following S phase; two regions from the same embryo are shown in which there is either a low concentration (unbound, left panels) or high concentration (bound, right panels) of the antibodies. Colored arrowheads indicate the same centrosomes in mitosis and then after centrosome separation; arrows highlight examples where binding of the antibody has perturbed the recruitment of Asl-GFP (2/2 embryos) or Polo-GFP (2/3 embryos) to new centrioles. Scale bars, 5 μm. (C) Polo-GFP (green) localization at newly separated centrioles in embryos expressing WT Sas-4-mKate2 (mKate2 was used as a red fluorescent tag due to its relatively short fluorescence maturation time compared to other red fluorescent proteins; see Supplemental Experimental Procedures) or Sas-4-mKate2-Thr200-STP-motif mutant forms (red; as indicated). Note that Polo-GFP localizes symmetrically at newly separated centriole pairs in embryos expressing WT Sas-4-mKate (arrowheads), however, Polo-GFP localization is strongly disrupted or absent from new centrioles in embryos expressing T200A, P201G, or S199G mutant forms of Sas-4-mKate2. Arrows highlight examples where the mutant fusion protein has perturbed the recruitment of Polo-GFP to new centrioles, while arrowheads indicate the unperturbed older centrioles in these embryos. Scale bars, 5 μm (left panel) and 2 μm (right panel). See also Figure S3.

When we injected the anti-Sas-4-pThr200 antibodies at higher levels, we noticed that the antibody perturbed the recruitment of Asl-GFP to new centrosomes at the end of mitosis (Figure 3A). This strongly argues (although it does not conclusively prove) that the antibody is specifically binding to the phospho-Sas-4-Thr200 epitope in embryos, rather than non-specifically binding to a different centrosomal protein, as the antibody can elicit the same very specific phenotype as mutating Thr200. Moreover, we noticed that high concentrations of antibody also appeared to block the recruitment of Polo-GFP to the newly converted mother centrioles without interfering with Polo-GFP localization at the older mother centriole (Figure 3B). Thus, remarkably, interfering with a single putative Polo-docking domain on Sas-4 appears to be sufficient to block the recruitment of Polo to new centrioles in embryos.

To test this possibility further, we assayed the effect of separately expressing three mutant forms of the Sas-4-Thr200-STP motif (S199G, T200A, and P201G) on Polo recruitment to new centrioles. Like the phospho-specific antibody, each of these mutant forms of Sas-4 strongly perturbed the recruitment of Polo-GFP to new centrioles (Figure 3C). Interestingly, however, neither the anti-Sas-4-pThr200 antibodies nor the Sas-4-Thr200-STP-motif mutants perturbed centriole disengagement at the end of mitosis (Figures 1B, S3E, and S3F). This suggests that centriole disengagement does not require Polo recruitment to the daughter centriole.

### Sas-4-Thr200 Is Required for Mitotic Centriole Conversion In Vivo

**Figure 4:**
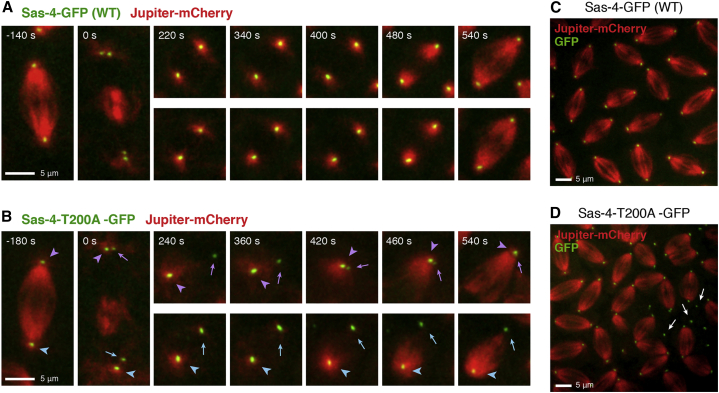
Sas-4-Thr200 Is Required for Centriole Conversion (A and B) Time-lapse images show the localization of Jupiter-mCherry (MT marker, red) in living embryos expressing WT Sas-4-GFP (A) or Sas-4-T200A-GFP (B) from injected mRNA (green, as indicated). (A) In embryos expressing WT Sas-4-GFP, centrioles separate at the end of mitosis (t = 0); both centrioles form centrosomes that nucleate robust MT arrays throughout S phase (t = 220–400 s) and later organize the mitotic spindle poles (t = 540 s). (B) In embryos expressing Sas-4-T200A-GFP, the old centrioles (arrowheads) organize a centrosome that nucleates MTs, but new centrioles (arrows) are unable to efficiently convert into functional MT-organizing centers (t = 240–540 s), leading to spindle abnormalities during mitosis (t = 540 s). (C and D) Images show metaphase-stage living embryos expressing WT Sas-4-GFP (C) or Sas-4-T200A-GFP (D) from injected mRNA (green, as indicated). Jupiter-mCherry (red) localization shows the mitotic spindles. Note that embryos expressing Sas-4-T200A-GFP contain multiple “unconverted” centrioles that do not participate in spindle formation; in these embryos abnormal, conjoined spindles are formed by the old centrosomes while the new centrioles float nearby in the cytoplasm (white arrows in D). Such spindle defects were observed in six of nine embryos injected with Sas-4-T200A-GFP, but in none of three embryos injected with WT Sas-4-GFP. Scale bars, 5 μm.

Polo and Asl both play an important part in mitotic PCM recruitment in flies (Conduit et al., 2014a, Conduit et al., 2014b, Dobbelaere et al., 2008, Sunkel and Glover, 1988, Varmark et al., 2007). We predicted, therefore, that although the expression of Sas-4-T200A does not block centriole disengagement, it should block the subsequent recruitment of PCM to the disengaged daughter centriole that cannot recruit Polo or Asl. In embryos expressing WT Sas-4-GFP, new centrioles formed centrosomes after they separated from their mothers (as judged by their ability to organize MTs), which invariably formed spindle poles during the next mitosis (Figures 4A and 4C). In contrast, in embryos expressing Sas-4-T200A-GFP many of the new centrioles failed to organize MTs after they separated from their mothers, and these subsequently failed to form spindle poles (Figures 4B and 4D). As a result, many of the spindles in these embryos shared spindle poles organized by the original mother centrioles, while the “unconverted” centrioles floated freely in the cytoplasm (arrows in Figure 4D). Thus, the Sas-4-T200A point mutation appears to block mitotic centriole conversion and subsequent centrosome assembly in embryos.

### The Sas-4-Thr200-STP Motif Is Essential for Early Embryonic Development

In these RNA injection experiments we had to express the Sas-4-GFP fusion protein in the presence of the endogenous (but unlabeled) Sas-4 protein, as mutant flies lacking Sas-4 are uncoordinated (unc) due to the lack of cilia and so cannot mate or lay embryos (Basto et al., 2006) (see schematic, Figure S4A). To test whether the Sas-4-Thr200-STP motif was essential for Sas-4 function in vivo, we generated stable transgenic lines expressing mCherry fusions to either WT or STP-motif mutant forms of Sas-4. These fusion proteins were all expressed at similar levels (Figures S4C and S4D), but whereas WT Sas-4-mCherry efficiently rescued the unc defect of *Sas-4* mutant flies, Sas-4-T200A-mCherry rescued this phenotype very poorly, indicating that the rescued flies still had significant cilia defects (Figure S4B).

**Figure 5:**
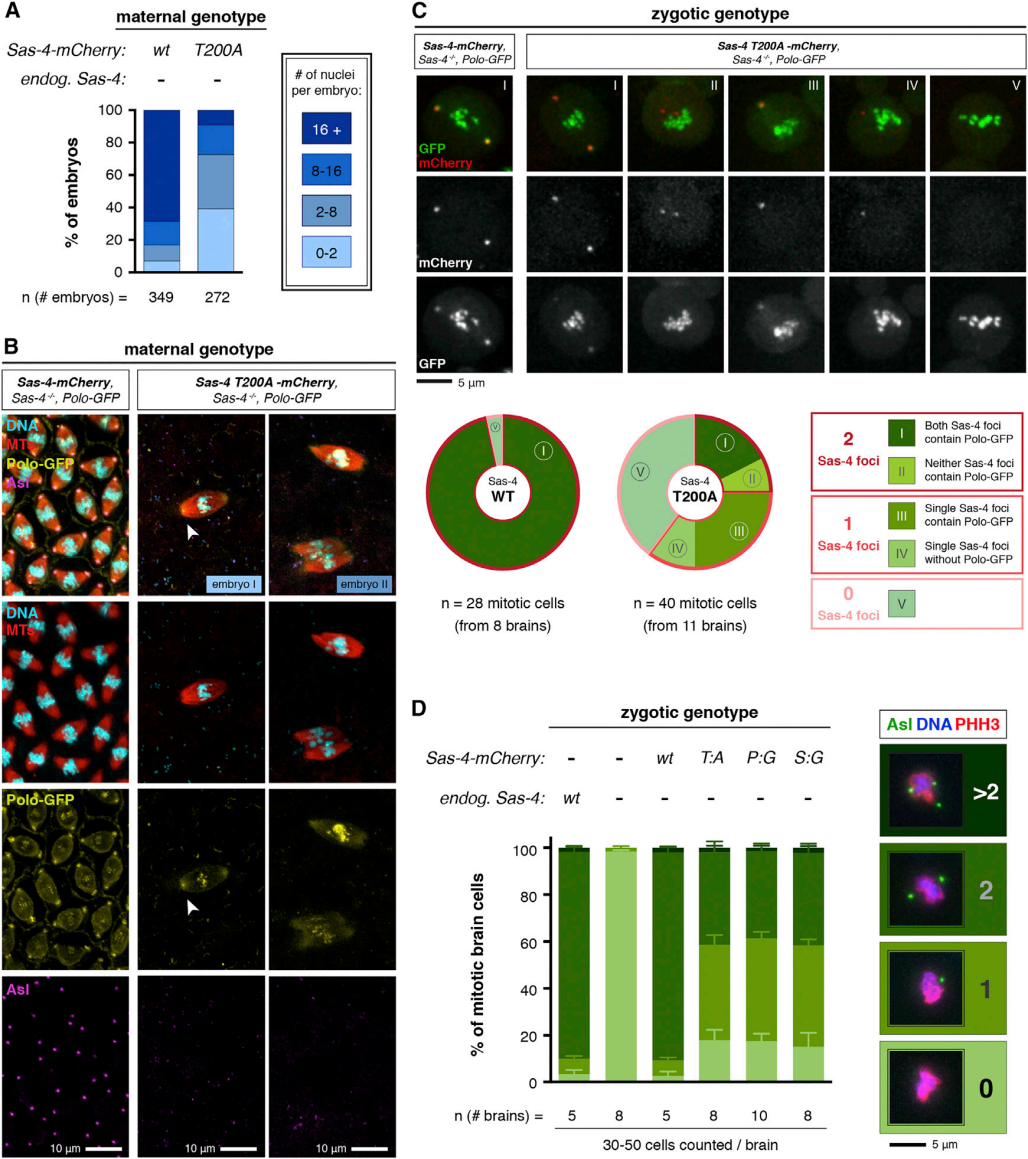
The Sas-4-Thr200-STP Motif Is Essential for Sas-4 Function In Vivo (A) Quantification of nuclei number in 0- to 2-hr-old *Sas-4* mutant embryos expressing endogenous levels of either Sas-4-mCherry (n = 349 embryos) or Sas-4-T200A-mCherry (n = 272 embryos). (B) Micrographs of typical embryos stained to reveal the distribution of MTs (red), nuclei (cyan), Polo-GFP (yellow), and the centrosomal marker Asl (magenta). Note that both Polo-GFP and Asl are enriched at the spindle poles in embryos expressing Sas-4-mCherry, while their localization is severely disrupted in embryos expressing Sas-4-T200A-mCherry, resulting in the formation of mitotic spindles that usually lack detectable centrosomes. The arrowhead in embryo I highlights a pole with a centrosome that contains some Polo and low levels of Asl; the spindles in embryo II do not detectably have centrosomes at their poles. Scale bars, 10 μm. (C) Micrographs show examples of living mitotic larval neuroblast cells co-expressing Polo-GFP with Sas-4-mCherry or Sas-4-T200A-mCherry. Graphs below show the percentage of mitotic cells that contain the indicated number of Sas-4 foci that are either Polo-GFP positive (darker green shading; categories I and III) or Polo-GFP negative (lighter green shading; categories II and IV); the percentage of cells without any detectable centrosomes is indicated by blue/green shading (category V). The percentages in each category are: Sas-4-mCherry: I, 96.43%; V, 3.57%; Sas-4-T200A-mCherry: I, 17.5%; II, 7.5%; III, 25%; IV, 10%; V, 40%. Scale bar, 5 μm. (D) Graph shows the quantification of Asl foci (typical examples of each class are illustrated in the micrographs) in mitotic (phospho-histone H3 [PHH3] positive) third instar larval brain cells of the indicated genotypes. Error bars indicate SEM. Scale bar, 5 μm. See also Figure S4.

Embryos laid by *Sas-4* mutant females rescued by WT Sas-4-mCherry developed normally, but embryos laid by mutant females rescued by any of the three mutated forms of Sas-4-mCherry (S199G, T200A, or P201G) failed to hatch as larvae (>1,000 scored for each genotype; data not shown). A detailed analysis of mutant embryos expressing Sas-4-T200A-mCherry revealed that they all arrested very early in development with only a few nuclei (Figure 5A). The spindles in these embryos were often disorganized, and of 18 optically sectioned spindle poles only seven had detectable Asl and Cnn staining (data not shown). We also expressed Polo-GFP in these embryos to allow us to follow Polo behavior. Of the 24 spindle poles we optically sectioned in these embryos, only five had detectable Asl or Polo staining (Figure 5B). Centrosomes are essential for early embryonic development in flies, and embryos lacking functional centrosomes arrest with a very similar phenotype to that we observe here (Stevens et al., 2007, Varmark et al., 2007). Thus, these observations support the conclusion from our embryo mRNA injection studies that the Sas-4-Thr200-STP motif is essential for proper centriole duplication and centrosome assembly in embryos.

### The Sas-4-Thr200-STP Motif Is Required for Efficient Polo and Asl Recruitment and Efficient Centriole Duplication and Centrosome Assembly in Somatic Brain Cells

The nuclear division cycles of early *Drosophila* embryos are extremely short and thus necessitate very rapid and efficient mitotic centriole conversion. We wanted to test whether the Sas-4-STP motif was important for centriole conversion during the much longer cell cycles in cell types other than embryos. We therefore examined the recruitment of Polo-GFP to centrosomes in living *Sas-4* mutant mitotic larval brain cells expressing either WT Sas-4-mCherry or Sas-4-T200A-mCherry. Almost all the mitotic cells expressing Polo-GFP and WT Sas-4-mCherry had two centrosomes that were strongly decorated with both fusion proteins (Figure 5C). In contrast, ∼40% of cells expressing Polo-GFP and Sas-4-T200A-mCherry had no detectable centrosomes, ∼35% had one centrosome, and only ∼25% had two centrosomes (as judged by the presence of Sas-4 foci). In the cells that had at least one centrosome, ∼30% of these Sas-4 foci contained no detectable Polo-GFP (suggesting these were not functional centrosomes but rather unconverted centrioles), while the rest had detectable Polo-GFP, although it was often only weakly localized (Figure 5C).

We obtained similar results when we analyzed the distribution of Asl in fixed *Sas-4* mutant brains expressing WT or Sas-4-Thr200-STP-motif mutant mCherry-fusion proteins (Figure 5D). All the brains rescued by the Sas-4-STP mutant proteins exhibited reduced centrosome numbers (as judged by the number of Asl foci), and, in those cells that contained centrosomes, Asl recruitment was often very weak (Figures 5D, S4E, and S4F). We note that somatic brain cells can occasionally form acentrosomal MT-organizing centers that contain Asl, but not Sas-4 (Baumbach et al., 2015), potentially explaining why the reduction in centrosome numbers in the mutants appears to be greater when we score centrosomes by counting Sas-4 foci (Figure 5C) than when we count Asl foci (Figure 5D). We conclude that Polo and Asl recruitment to centrioles, and centriole duplication and centrosome assembly, are strongly perturbed in *Sas-4* mutant brain cells that are rescued by Sas-4-Thr200-STP-motif mutants.

## Discussion

It has long been known that newly formed daughter centrioles are unable to duplicate during the S phase in which they were born, even under conditions that allow mother centrioles to proceed through multiple rounds of duplication (Cunha-Ferreira et al., 2009, Kleylein-Sohn et al., 2007, Loncarek et al., 2008). How exactly daughter centriole duplication in S phase is prevented is unclear, but it has recently been shown that Polo/Plk1 modifies the daughter centriole during mitosis in some way that allows it to subsequently duplicate and form a centrosome (Wang et al., 2011). This process has previously been termed centriole-to-centrosome conversion (Fu et al., 2016, Izquierdo et al., 2014, Wang et al., 2011), although we prefer the term “mitotic centriole conversion” because, although in fly embryos the converted centrioles almost immediately form centrosomes and duplicate, in other cell types the converted centrioles may only duplicate or form a centrosome much later in the next cell cycle (when the cells eventually enter S phase or M phase, respectively). Thus, mitotic centriole conversion generates a centriole that is *competent* to both duplicate and form a centrosome, although these events may occur independently and sometime after the initial conversion event, depending on the cell type.

In flies, the recruitment of Asl to the new centriole during mitosis appears to be a critical event in centriole conversion, as Asl incorporation ultimately allows centrioles to both duplicate and recruit mitotic PCM (Conduit et al., 2014b, Novak et al., 2014). Importantly, a fraction (∼50%) of Asl is incorporated into centrioles stably (Novak et al., 2014), meaning that once daughter centrioles have passed through their first mitosis, they no longer have to pass through mitosis again to acquire Asl. In this way, Asl incorporation acts as a “primary license” whose acquisition during mitosis is required to allow new centrioles to duplicate and form a centrosome for the first time (Novak et al., 2014). Thus, mother centrioles do not normally reduplicate during S phase because they are engaged to their daughters (Loncarek et al., 2008, Tsou and Stearns, 2006a, Wong and Stearns, 2003), while daughter centrioles cannot duplicate at all because they lack Asl and so cannot recruit Sak/Plk4 (Dzhindzhev et al., 2010, Novak et al., 2014).

**Figure 6:**
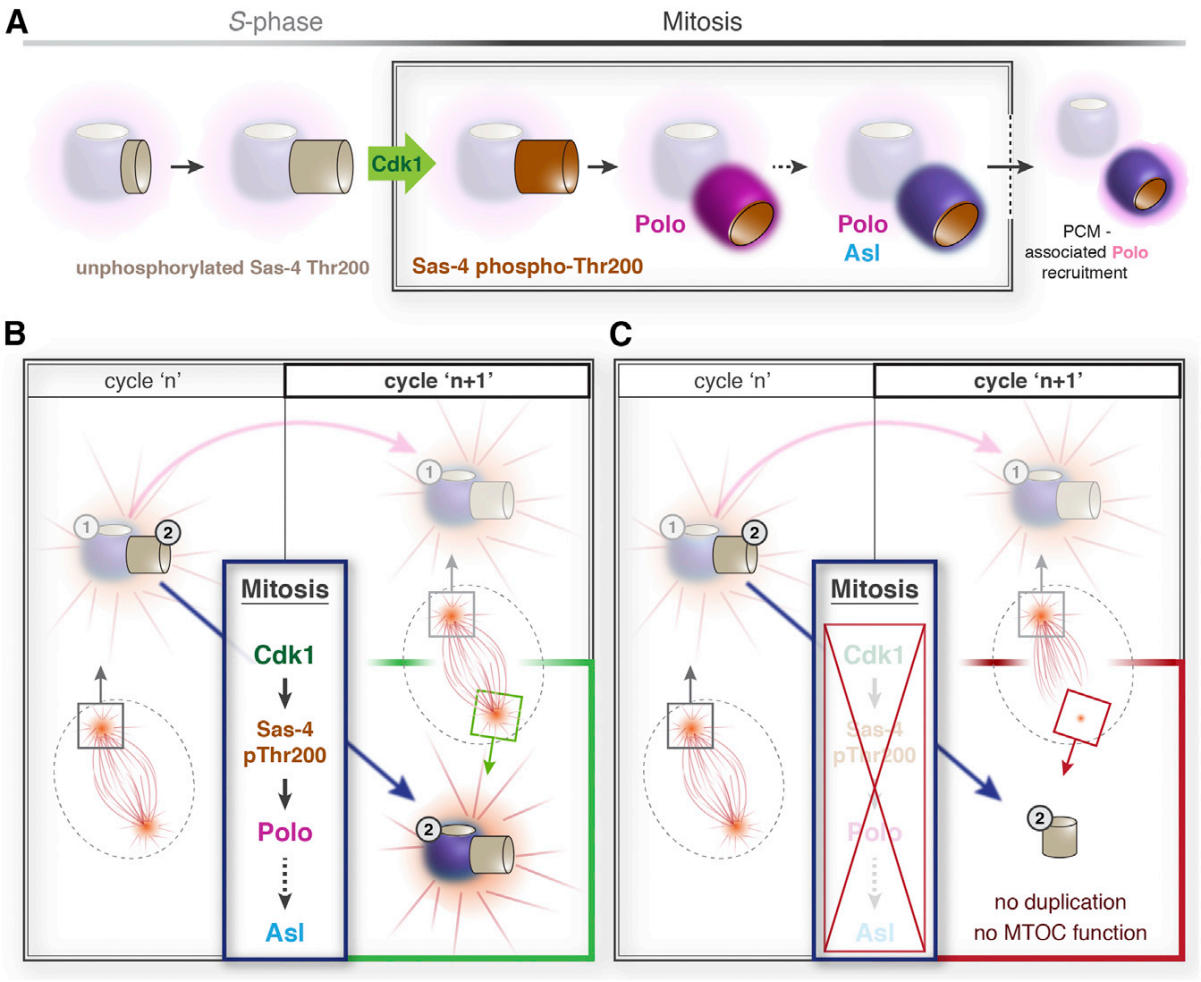
Model (A) Schematic model illustrates how Sas-4 (light gray) is incorporated into new centrioles during S phase and is phosphorylated on Thr200 (brown) by Cdk1 as cells enter mitosis, thus allowing the recruitment of Polo (magenta) and then Asl (blue). The recruitment of Asl allows the new centriole to recruit PCM (which also contains Polo and so is indicated by the magenta cloud around the fully converted centriole). The mother centriole and its associated PCM are indicated semi-transparently. (B and C) Schematics summarize the importance of the Cdk1- and Polo-regulated conversion of new centrioles to duplication-competent MT-organizing centers (MTOC) during mitosis. (B) Daughter centrioles (indicated as “2” in cycle “n”) are phosphorylated by Cdk1 on Sas-4 Thr200 during mitosis, allowing the initial recruitment of Polo, and ultimately Asl, to these centrioles. This mitotic cascade allows these new centrioles to template centriole duplication as well as organize PCM during the following cycle (cycle “n+1”), and thus ensures the formation of sufficient functional centrosomes during successive cell generations. (C) New centrioles that are not phosphorylated on Sas-4-Thr200 fail to convert into functional centrosomes, which can result in spindle abnormalities during the following cell cycle (cycle “n+1”), and potentially complete centrosome depletion during later cell divisions. Note that successful mitotic centriole conversion affects the centrosomal numbers and function in future cell cycles, not the cycle in which the conversion itself happens. Centrioles are only affected by this Cdk1-Polo-Asl cascade during their first mitosis: older centrioles (indicated as “1”) that were converted in previous cycles are no longer dependent on this regulatory process to allow their continuing duplication and function.

In this study we have identified a Polo/Plk1-dependent mechanism that is required to recruit Asl to newly formed centrioles during mitosis, and we have shown that this mechanism appears to be initiated by the recruitment of Polo to the daughter centriole by the Cdk1-dependent phosphorylation of Sas-4 (Figure 6). Sas-4 is a conserved centriole protein that is essential for centriole duplication in flies (Basto et al., 2006), and we previously showed that it is involved in recruiting Asl to newly formed centrioles during mitosis (Conduit et al., 2014b, Novak et al., 2014). Cdk1 can phosphorylate the Sas-4-Thr200-STP motif to create a Polo-docking site in vitro, and an analysis of several STP-motif mutants reveals that mutations that disrupt Cdk1 phosphorylation or PBD binding in vitro invariably perturb both Polo and Asl recruitment to the new centriole in vivo, leading to severe defects in centriole duplication and centrosome assembly. Moreover, antibodies that specifically recognize the phosphorylated Sas-4-Thr200-STP motif in vitro localize to centrosomes during mitosis and can also specifically block the recruitment of Polo and Asl to newly disengaged daughter centrioles.

The simplest interpretation of this collective data is that the Polo-docking site on Sas-4 directly recruits Polo to the new centriole, although we cannot exclude the possibility that this Sas-4-Thr200-STP motif may not bind Polo in vivo and actually performs some other unknown function that indirectly is required for Polo recruitment. Nevertheless, our findings now directly implicate Cdk1 kinase in promoting the centriole duplication cycle through the direct phosphorylation of a centriolar protein, and provide an intriguing molecular insight into the mechanism that likely regulates the primary recruitment of the crucial cell-cycle regulator Polo/Plk1 kinase to newly formed centrioles in *Drosophila*.

In fly embryos the Sas-4-Thr200-STP motif appears to be essential for centriole conversion. In somatic fly cells this motif appears to be important, but it is not essential; STP-motif mutations perturb Asl and Polo recruitment to centrosomes, and centrosome numbers are dramatically reduced, but centrosome duplication and centrosome assembly are not abolished. These data suggest that the Sas-4-Thr200-STP motif plays a critical role in promoting efficient centriole conversion rather than being an indispensable component of this process. We speculate that when the enrichment of Polo at daughter centrioles is perturbed by Sas-4-Thr200-STP mutations then other, suboptimal, Polo-docking sites within the daughter centriole, or the Polo pool present in the cytoplasm or in the PCM surrounding the neighboring mother centriole, can still initiate the molecular cascade leading to the conversion of the daughter centriole, although with far lower efficiency. Such a reduction in efficiency appears sufficient to block conversion during the rapid divisions in the syncytial embryo (where daughters must be converted in just a few minutes), but is not sufficient to completely block this process in brain cells, which have much longer cell-cycle times and thereby a greater time window in which to complete centriole conversion.

Surprisingly, disrupting the recruitment of Polo to daughter centrioles did not appear to alter the efficiency of centriole disengagement, even though Polo/Plk1 is of critical importance in this process (Loncarek et al., 2010, Shukla et al., 2015, Tsou et al., 2009). This demonstrates that the recruitment of Polo to daughter centrioles, at least in flies, does not play a key role in timing centriole disengagement; instead the pool of Polo on the mother centriole (or in the cytoplasm) is sufficient to drive this event.

The recruitment of Polo/Plk1 to daughter centrioles during mitosis is likely a key regulatory event in the centrosome cycle. It is perhaps surprising, therefore, that this recruitment relies so heavily on a single phosphorylation site on a single protein. We suspect that the phosphorylation of Sas-4-Thr200 is only required for the initial binding of Polo to new centrioles: once recruited, Polo probably phosphorylates several other nearby proteins to create further Polo-docking sites, thereby driving robust Polo recruitment that ultimately influences many aspects of centriole and centrosome function. How Polo docking to Sas-4 might enable Asl recruitment to daughter centrioles is unclear, but an attractive possibility is that Polo subsequently phosphorylates either Asl or other sites on Sas-4 to increase the efficiency of the previously characterized and conserved direct interaction between Sas-4 and Asl (Cizmecioglu et al., 2010, Dzhindzhev et al., 2010, Hatch et al., 2010). An alternative, and not mutually exclusive, possibility is that the centriole proteins Cep135 and Ana1 are phosphorylated by Polo, as both proteins also help to recruit Asl to centrioles (Fu et al., 2016), although there is some evidence that Ana1 may primarily help to generally maintain Asl at all mother centrioles, rather than specifically help to initially recruit Asl to new mother centrioles (Saurya et al., 2016). Future studies will doubtless identify the crucial substrates of Polo that allow Asl to be recruited to new centrioles during mitosis.

## Experimental Procedures

### Transgenic *Drosophila* Lines

The Asl-mCherry, Sas-4-mCherry (Conduit et al., 2014b), Sas-4-GFP (Novak et al., 2014), Asl-GFP (Blachon et al., 2008), Polo-GFP (Buszczak et al., 2006) and Jupiter-mCherry (Callan et al., 2010) lines used in this study have been described previously. In all experiments, Asl-mCherry or Asl-GFP was expressed at near-endogenous levels in the *asl*^*B46*^ (Baumbach et al., 2015) homozygous mutant background. Sas-4-GFP (when supplied transgenically, not in mRNA injection experiments) was expressed in the *Sas-4*^*S2214*^ (Basto et al., 2006) homozygous mutant background. To generate Sas-4-T200A-mCherry, Sas-4-P201G-mCherry, and Sas-4-S199G-mCherry lines, we introduced the respective point mutations into the Sas-4-mCherry P element transformation vector (Conduit et al., 2014b) using a QuikChange II XL Site-Directed Mutagenesis Kit (Agilent Technologies). The transgenic lines were generated by the Fly Facility in the Department of Genetics, University of Cambridge. Sas-4-mCherry, Sas-4-T200A-mCherry, Sas-4-P201G-mCherry, or Sas-4-S199G-mCherry were analyzed in the *Sas4*^*S2214*^/*Df*(*3R*)*BSC221* genetic background. OregonR was used as the WT control.

### RNA Synthesis and Microinjection

In vitro RNA synthesis was performed using a T3 mMESSAGE mMACHINE kit (Ambion) and RNA was purified using an RNeasy MinElute kit (Qiagen). All RNA constructs were injected at a concentration of 2 mg/ml into 0- to 30-min-old embryos. Sas-4-GFP constructs were injected into either *Asl-mCherry*, *asl*^*B46*^ or *Jupiter-mCherry* embryos (both lines are homozygous for the WT *Sas-4* allele), and Sas-4-mKate2 constructs were injected into *polo-gfp*(*Trap*)/*TM3* embryos (also homozygous for the WT *Sas-4* allele). Microinjected embryos were incubated at 22°C and imaged after 60–120 min (when GFP fusions were injected) or after 120–150 min after injection (when mKate2 fusions were injected; as the fluorescence maturation time of mKate2 appears significantly longer than that of GFP in flies), always within the syncytial blastoderm stage of development. Live imaging was performed using the spinning-disc confocal system described below.

### Live Imaging

Living syncytial blastoderm stage embryos were imaged on a PerkinElmer ERS spinning-disc confocal system on a Zeiss Axiovert microscope, using a 63 × 1.4NA oil-immersion objective. Thirteen confocal sections were collected from each embryo (0.5-μm steps) every 20 s.

Living third instar larval brains were also imaged on the spinning-disc confocal system specified above. Twenty-one confocal sections (0.5-μm steps) were collected of mitotic neuroblasts every 50 s.

### Quantitative Analysis of Living Embryos and Brains

Asl-mCherry and Polo-GFP localization in embryos injected with WT or mutant *Sas-4* RNA constructs was scored blind (movies were renamed and mixed post acquisition, and the entire dataset generated for this study was scored blindly at the same time). Embryos in which multiple new centrosomes lacked any detectable levels of Asl-mCherry were classified as showing defective Asl-mCherry recruitment, while in the case of Polo any embryos in which multiple centrosome pairs displayed strongly asymmetric Polo-GFP levels were counted as showing defective Polo-GFP recruitment. Embryos were scored qualitatively this way, and fluorescence levels were not quantified due to the variable nature of the RNA injection process. In embryos injected with anti-Sas-4-pThr200, Asl-GFP or Polo-GFP localization was compared within each embryo on the side closest to the injection site (centrosomes bound by antibody) and the side furthest away (unbound centrosomes). Timing of centrosome separation was also assessed this way in Sas-4-GFP embryos injected with anti-Sas-4-pThr200.

Quantification of Sas-4-mCherry and Polo-GFP foci in living larval brains was also performed blind. Movies of mitotic larval neuroblasts (identified by clustered, Polo-GFP-labeled kinetochores) were renamed and mixed post acquisition, and the entire dataset was scored blindly at the same time.

### Fixed Analysis of Embryos and Larval Brains

0- to 2-hr-old embryos were fixed and stained as described previously (Stevens et al., 2009). Guinea-pig anti-Asl (Roque et al., 2012) and mouse anti-α-tubulin (Sigma) primary antibodies were used at 1:500 dilution to immunostain embryos. GFP-Booster (atto 488, ChromoTek), Alexa Fluor anti-mouse 568, and anti-guinea-pig 647 (Life Technologies) were used at 1:500 dilution as secondary antibodies. Hoechst 33258 (Life Technologies) was used at 1:5,000 dilution to stain DNA. Images of fixed embryos presented in this study were collected using an Olympus confocal microscope (FV1200 IX83; Olympus) with Fluoview software, using a 60 × 1.3NA silicon oil-immersion Super Apochromat lens (UPLSAPO 60XS). Nineteen confocal sections were collected (0.2-μm steps). Nuclei numbers were quantified in fixed embryos using a Zeiss Axioskop 2 microscope with a 10 × 0.3NA dry objective.

Third instar larval brains were dissected, fixed, squashed, and stained as described previously (Stevens et al., 2009). The following primary antibodies were used at 1:500 dilution to immunostain brains: rabbit anti-phospho-Histone3 (Cell Signaling), rat anti-Asl (Franz et al., 2013), guinea-pig anti-Cnn (Lucas and Raff, 2007). Alexa Fluor anti-rat 488, anti-rabbit 568 and anti-guinea-pig 647 (Life Technologies) were used at 1:500 dilution as secondary antibodies. DNA was stained with Hoechst 33258 (1:5,000 dilution). All samples were blinded following slide preparation, prior to imaging and quantification. The samples were imaged and centrosome numbers were counted on a Zeiss Axioskop 2 microscope with Metamorph software, using a 63 × 1.25NA oil-immersion objective. Eleven confocal sections were collected (0.2-μm steps). Samples were unblinded after the quantification was completed for the full dataset. Percentage values were calculated for each brain (each datapoint represents a brain). Statistical comparisons of centrosome frequencies (shown in Figure S4F) were performed using the Mann-Whitney test (for pairwise comparisons) or the Kruskal-Wallis test (for simultaneous comparison of three or four genotypes).

### In Vitro Cdk1 Kinase Assay

Synthetic peptides (50 μM final concentration) were incubated with 40 units of recombinant human Cdk1-cyclin B (New England Biolabs) in 1× NEBuffer for Protein Kinases (New England Biolabs) with 100 μM cold ATP and 5 μCi γ-[^32^P]ATP in a reaction volume of 20 μl. Reactions were incubated at 30°C for 30 min, then terminated by the addition of 10 μl 7.5 M guanidine-hydrochloride. 1.2 μl of each reaction was spotted onto an avidin-coated membrane (SAM^2^ biotin capture membrane, Promega). After air-drying, the membrane was rinsed once with 2 M NaCl, then incubated for 3 × 2 min in 2 M NaCl, 4 × 2 min in 2 M NaCl + 1% H_3_PO_4_, rinsed twice in distilled water, and air-dried at room temperature for 1 hr. The dried membrane was exposed to an autoradiography film (Carestream BioMax MR) overnight at −80°C.

### In Vitro GST-Plk1 PBD Binding Assay

Streptavidin beads (30 μl/reaction; Dynabeads MyOne Streptavidin T1, Life Technologies) were washed three times with Binding Buffer (50 mM Tris [pH 8.0], 150 mM NaCl, 1 mM EDTA, 1 mM DTT, 1× protease inhibitor cocktail [Roche], 1× phosphatase inhibitor cocktail [Sigma]) and incubated with a 10-fold molar excess of biotinylated peptide in 1 ml of Binding Buffer for 30 min at room temperature. For analysis of the *Drosophila* Sas-4-Thr200-STP motif, the synthetic phosphopeptides and their non-phosphorylated counterparts were coupled directly to the beads. For analysis of the human CPAP-Thr616-STP motif, the synthetic non-phosphorylated peptides were pre-incubated in 1× NEBuffer for Protein Kinases (New England Biolabs) and 200 μM ATP (New England Biolabs) with or without 120 units of recombinant human Cdk1-cyclin B (New England Biolabs) in a final volume of 60 μl (3 nmol peptide/reaction) for 30 min at 30°C before coupling to the streptavidin beads as described above (the entire reaction volume was added to the beads together with 1 ml of Binding Buffer). This approach was taken because synthetic production of the phosphorylated version of the human CPAP-Thr616-STP peptide could not be completed by the manufacturer within the required time frame. The beads were rinsed four times by resuspension in 200 μl of Capture Buffer (50 mM Tris [pH 8.0], 150 mM NaCl, 1 mM EDTA, 1 mM DTT, 1× protease inhibitor cocktail [Roche], 1× phosphatase inhibitor cocktail [Sigma], 0.01% Tween 20, 0.01% BSA]), then incubated with 1 ml of PBD solution (0.1 μM recombinant human GST-Plk1-PBD [Sigma; #SRP0360, GST-Plk1 aa 367–603] in 50 mM Tris [pH 8.0], 150 mM NaCl, 1 mM EDTA, 2 mM DTT, 1× protease inhibitor cocktail [Roche], 1× phosphatase inhibitor cocktail [Sigma], 0.01% Tween 20, 0.01% BSA) for 3 hr at 4°C. The beads were rapidly washed four times in 250 μl of Wash Buffer (50 mM Tris [pH 8.0], 150 mM NaCl, 1 mM EDTA, 2 mM DTT, 1× protease inhibitor cocktail [Roche], 1× phosphatase inhibitor cocktail [Sigma], 0.1% Tween 20) then resuspended and boiled in 30 μl of SDS loading dye. 2 × 1.5 μl of each sample was spotted onto nitrocellulose membranes (Bio-Rad). The membranes were air-dried at room temperature and incubated in milk solution (PBS + 4% milk + 0.1% Tween 20) for 1 hr. One of the two membranes was probed using rabbit anti-GST antibody (Abcam; 1:500 dilution) and anti-rabbit-horseradish peroxidase (HRP) (GE Healthcare; 1:3,000 dilution) for GST-Plk1-PBD detection, and the other membrane was probed with streptavidin-HRP (Thermo Scientific; 1:3,000 dilution), as the peptide loading control. Three technical repeats were performed.

### Rescue Experiments

*Sas-4-mCherry*, *Sas-4-T200A-mCherry*, *Sas-4-P201G-mCherry*, and *Sas-4-S199G-mCherry* transgenes were crossed into the *Sas4*^*S2214*^/*Df*(*3R*)*BSC221* genetic background for all rescue experiments. All flies analyzed (embryonic, larval, or adult phenotypic assessments) were homozygous for the transgene they carried. For assessment of the importance of Sas-4-Thr200 in Polo recruitment, *polo-gfp*(*Trap*) was crossed into each of the *Sas-4-mCherry, Sas4*^*S2214*^/*Df*(*3R*)*BSC221* and *Sas-4-T200A-mCherry*, *Sas4*^*S2214*^/*Df*(*3R*)*BSC221* genetic backgrounds (Polo-GFP was only expressed in the rescue experiments where its presence is indicated in the respective figure panel).

Embryonic phenotypes were assessed in the progeny of rescued females that were mated with OregonR males. Several hundred such embryos were collected from mutant females rescued by each *Sas-4-mCherry*, *Sas-4-T200A-mCherry*, *Sas-4-P201G-mCherry*, or *Sas-4-S199G-mCherry*. None of the embryos laid by mothers rescued by any of the three STP mutant constructs hatched as larvae and 100% of these embryos arrested before gastrulation, as judged by their homogeneous white color even following >7 days of incubation at 25°C. Detailed immunofluorescence analysis was then performed on the embryos laid by *Sas-4-T200A-mCherry*, *Sas4*^*S2214*^/*Df*(*3R*)*BSC221* mothers (both with or without co-expression of Polo-GFP) to confirm that eggs were fertilized and to analyze the nature of the early embryonic arrest.

## Author Contributions

Z.A.N. designed and performed the experiments and wrote the manuscript. A.W. performed some of the immunofluorescence imaging. L.G. optimized the use of the mKate2 fluorescent tag in the RNA injection screen system. J.W.R. designed experiments and wrote the manuscript.

## Supplemental Information

Document S1. Supplemental Experimental Procedures and Figures S1–S4

Document S2. Article plus Supplemental Information
