# Supplementary material for: Cdk1 Phosphorylates Drosophila Sas-4 to Recruit Polo to Daughter Centrioles and Convert Them to Centrosomes
Source: Dev Cell. 2016 Jun 20;37(6):545–57. doi: 10.1016/j.devcel.2016.05.022 (PMC4918730; doi:10.1016/j.devcel.2016.05.022)
Supplement: Document S1. Supplemental Experimental Procedures and Figures S1–S4 [file mmc1.pdf]

Developmental Cell, Volume *37*

Supplemental Information

Cdk1 Phosphorylates *Drosophila* Sas-4 to Recruit Polo to Daughter Centrioles and Convert Them to Centrosomes

Zsofia A. Novak, Alan Wainman, Lisa Gartenmann, and Jordan W. Raff

Inventory of Supplemental Materials:

- **Figure S1,** related to Figure 1. Identifying mutations in Sas-4 that interfere with the loading of Asl onto new centrioles.
- **Figure S2,** related to Figure 1. Sas-4-Thr200 is required to load Asl onto new centrioles.
- **Figure S3,** related to Figures 2 & 3. The Sas-4-Thr200-STP-motif is required to load Polo onto new centrioles.
- **Figure S4,** related to Figure 5. Analysis of Sas-4-Thr200-STP-motif mutant transgenic flies.
- **Supplemental Experimental Procedures**
- **Supplemental References**

Supplemental Figures

A

***Drosophila melanogaster* Sas-4:**

1 - MQEAGESPVGMPLSQEAIAQRLAALSRWQDEQKRLLQERQSNHRVLLGLEQRNMYKMLGLLHQETESHENSVLEEWDEEHSIQMPRLAADPDEHEPEISMADPQPAKPKRPFLRRGEGLKQRFKI **- 125**

126 - NPDQLRLENLPRYKFANAHPQFRTPQMKKGILKKQVKSPPNPAPPPAPKPSAQLDDLNEQRFQQMLIGKNACSSTPDLKSSYASSTTASSTSPRVRFVEQKSRAGQTTHADDEASSEASPMTGVC **- 250**

251 - WAKVLDTQSIKPAQIQRRSAQIRVEDDSNVSIFELLEQKATEGNIDMNSSCIRTFMARKDQRRRVADDSDHIVVTQQVRQMRLQPQVDQMLVQELQEGDEEDTEPSSDQTLTMTPIQVGNTKVRV **- 375**

376 - RFSDSNDTHEYSDATSLNDGSNIQLFEQFKSALFQALEQKKKSSPSKESDEPITKDLQEKANLVRTRLEELETEIATFKEQNAQLLRLRQQHELEKAKCTQDHMEAMERVHDEKIQAEIYLHDER **- 500**

501 - MKIEEERRKFEQQMRLQKSNANSKEKKEIAALKQEVEGLQLQLKQKEQAHVSAQARLRAQLRASEKEQRNYRDEIELLRKENKRLEQELVKIGRENNSKMLQEINRNIARLAPKVLPSATMSDIL **- 625**

626 - DENGRRTQSLDGTAGKPAKQREAQNRRQSSGSAQVRSRSRSLSRNKKTPYALDESYASSSSVESEEVQPPVTAPKAATPPPAANSSSDFKREITNADGSKDIWYPNGNLKKISADGMNLRMLYFN **- 750**

751 - KDIKETNIREGTVKYYYAETNTWHTSYLDGLEILEFPNGQTEHRRKDGTVEIHFPNNSIKIVDPSDTEKLEEWRYADGTHLVQLRNGDKILNLPNGQKEIHTKLNKRREYPDGTVKLVYPDGSQE **- 875**

876 - TRYSNGRVRLKDKDGKLIMDTDYAKY **- 901**

B

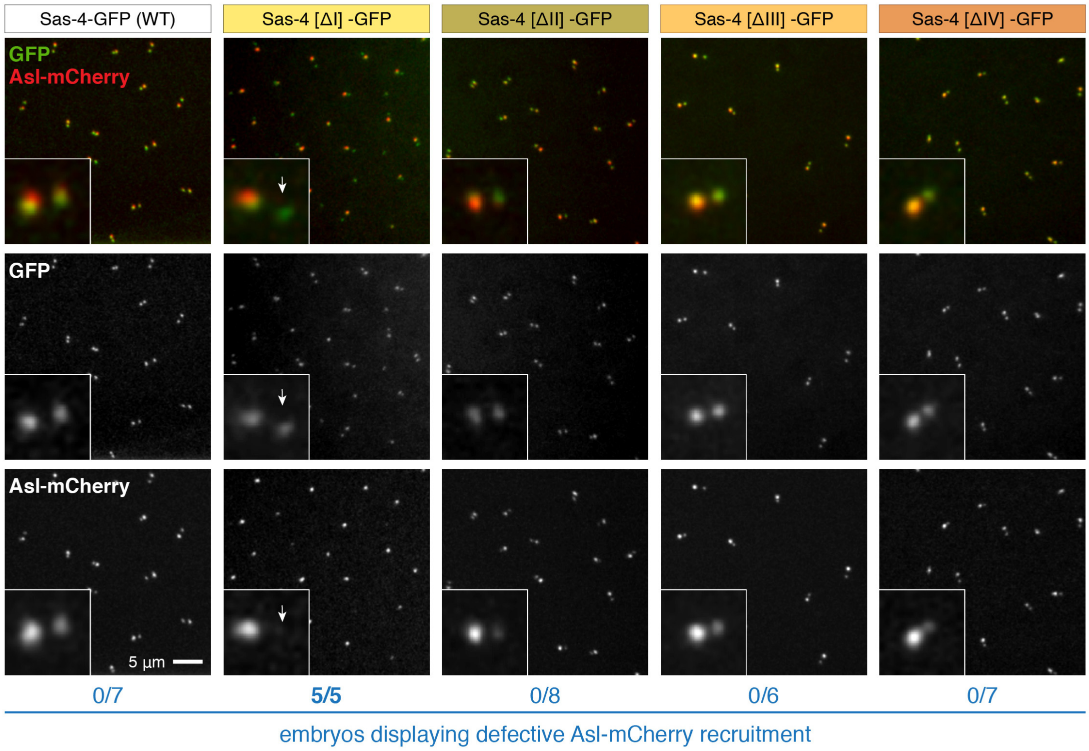

Figure S1, related to Figure 1.

Identifying mutations in Sas-4 that interfere with the loading of Asl onto new centrioles. A) Amino acid sequence of *Drosophila melanogaster* Sas-4, indicating the Asl-interacting region (*grey*) (Dzhindzhev et al., 2010) and the four regions of interest we identified that contain multiple Ser/Thr residues that are highly conserved across fly species (I-IV, highlighted in different colours). **B)** Micrographs illustrate Asl-mCherry (*red*) localization at newly separated centrioles in living embryos expressing full length Sas-4-GFP or the GFP-fusion of one of the four deletion constructs (*green*), as indicated; arrows highlight new centrioles that have separated from their mothers, but have not incorporated Asl-mCherry. The deletion of regions II, III or IV does not detectably interfere with Sas-4 localisation or Asl incorporation; deletion I does not detectably interfere with Sas-4 localisation, but dramatically perturbs Asl incorporation at new centrioles.

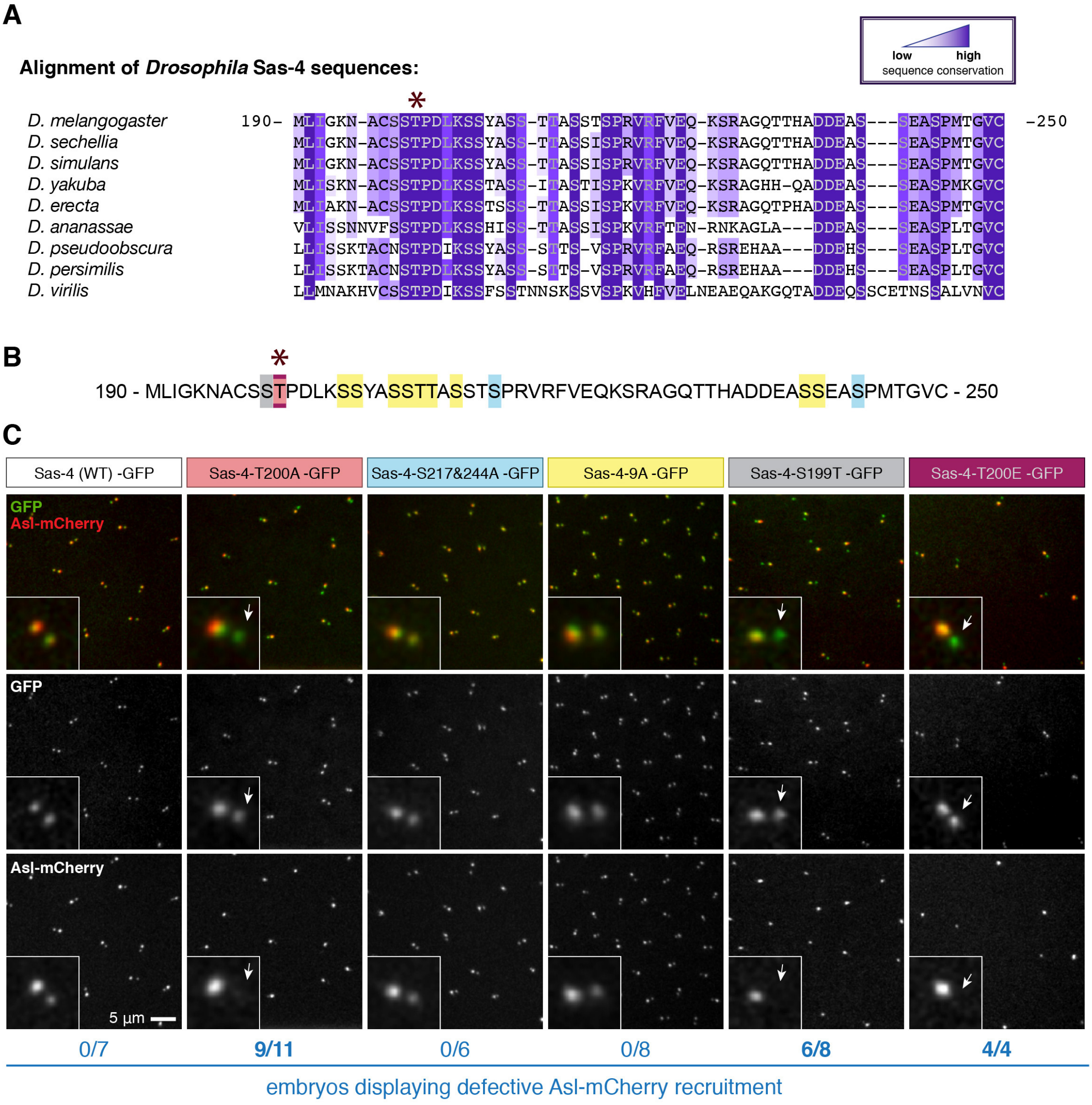

Figure S2, related to Figure 1.

Sas-4-Thr200 is required to load Asl onto new centrioles. A) Amino acid sequence alignment of Sas-4 region I from several *Drosophila* species (alignment was performed using the full protein sequence from each species); Thr200 is highlighted by an *asterisk*. **B)** The amino acid sequence of Sas-4 region I from *Drosophila melanogaster* highlighting three highly conserved putative Cdk phosphorylation sites—Thr200 (*pink/maroon; asterisk*), Ser217 and Ser244 (*blue*)—and 9 other conserved Ser/Thr residues (*yellow*). **C)** Micrographs show Asl-mCherry (*red*) localization at newly separated centrioles in living embryos expressing either full length Sas-4-GFP or the indicated mutated forms of Sas-4-GFP (*green*) in which either Thr200 (*pink label*), Ser217 and Ser244 (*blue label*), or all 9 of the other conserved Thr/Ser residues highlighted in (B) (*yellow label*), have been mutated to Ala. Note that, of these three constructs, only the T200A mutation appears to perturb the loading of Asl onto new centrioles (arrows). Asl-mCherry localization appeared normal in embryos injected with Sas-4-S217&244A-GFP or Sas-4-9A-GFP, but was disrupted in embryos injected with Sas-4-T200A-GFP. Micrographs also show that each the Sas-4-GFP S199T (*grey label*) and the T200E mutation (*maroon label*) disrupt the recruitment of Asl-mCherry to new centrioles similarly to the T200A mutation (arrows).

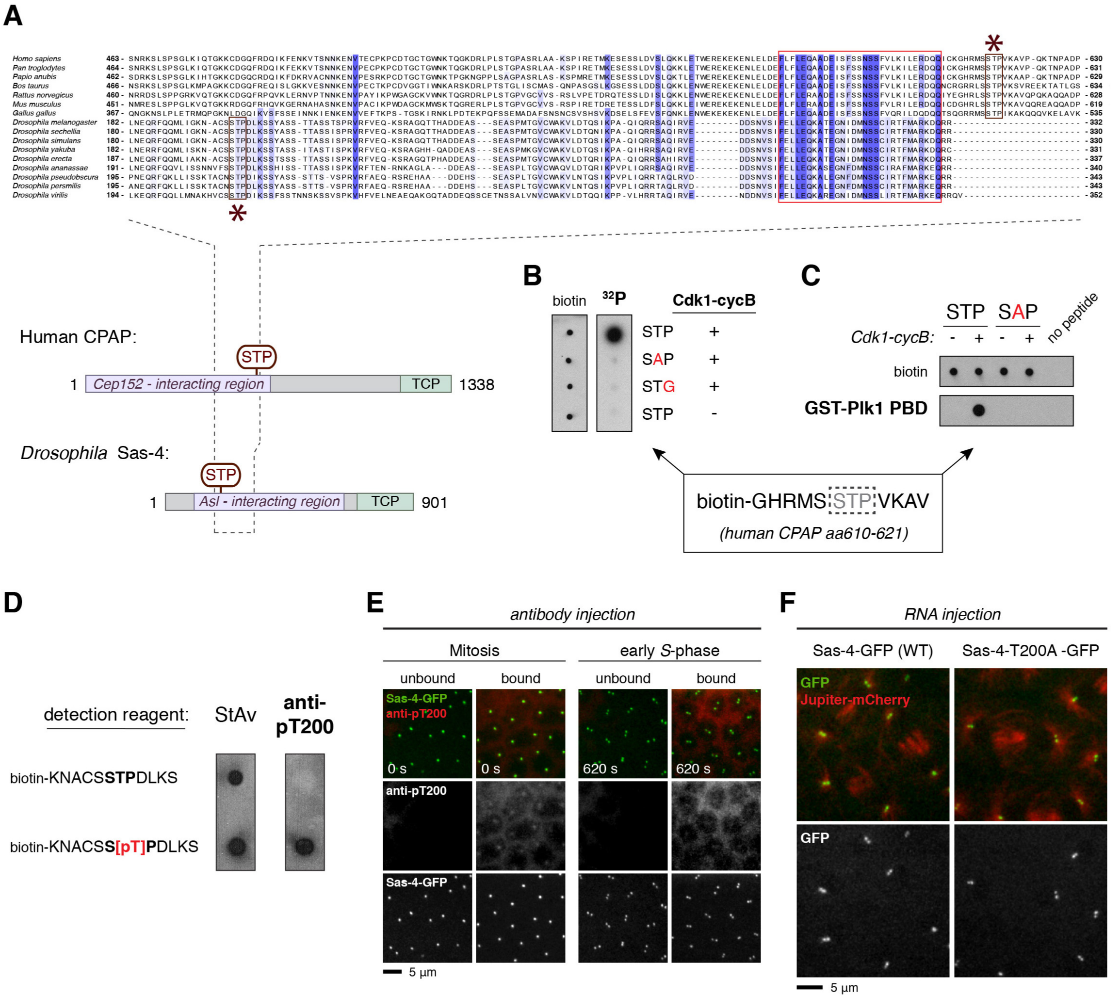

<u>Figure S3, related to Figures 2 & 3.</u>

The Sas-4-Thr200-STP-motif is required to load Polo onto new centrioles. A) A schematic representation of human CPAP and *Drosophila* Sas-4 highlighting the conserved TCP domain (*green*) that interacts with STIL/Ana2 (Cottee et al., 2013; Hatzopoulos et al., 2013) and the region shown to interact directly with Cep152/Asl (*purple*) (Cizmecioglu et al., 2010; Dzhindzhev et al., 2010; Hatch et al., 2010). This latter region is not generally well conserved, and the dotted lines delineate regions where an amino acid sequence alignment between a number of vertebrate and *Drosophila* species is shown below. These regions contain a short stretch of reasonable homology between the fly and vertebrate proteins (*red box*); the conserved Sas-4-Thr200-STP-motif (*asterisk*) in flies is located just upstream of this region, while the vertebrate sequences contain a conserved STP motif (*asterisk*) located slightly downstream from this region. **B)** *In vitro* assay of Cdk1/Cyclin B-dependent phosphorylation of the human CPAP-Thr616-STP-motif. The dot blot shows the loading of the biotinylated peptides (left panel); the autoradiogram shows the incorporation of ^{32}P (right panel). **C)** *In vitro* assay of the human CPAP-Thr616-STP-motif binding to recombinant GST-Polo-Box protein. The peptides were pre-phosphorylated with Cdk1/Cyclin B prior to incubation with GST-Polo-Box as indicated (see Experimental Procedures). The dot blots show the loading of peptide (top panel), and the binding of GST-Polo-Box-domain (bottom panel). **D)** Western blots confirm the specificity of the anti-Sas-4-pThr200 antibody: this antibody recognizes a peptide containing the Sas-4-Thr200 region only if Thr200 is phosphorylated (right panel). Streptavidin-HRP (StAv) was used to confirm the equal loading of the biotinylated peptides (left panel). **E)** Anti-Sas-4-pThr200 antibodies do not block centriole disengagement at the end of mitosis. Images show the timing of centrosome separation in a transgenic Sas-4-GFP (*green)* embryo that has been injected with anti-Sas-4-pThr200 (*red*). Two regions from the same embryo are shown, in which there is either a low concentration (unbound) or high concentration (bound) of antibody, and the embryo is shown during mitosis (left panels) and the following S-phase (right panels). Note that binding of the antibody to centrosomes during mitosis does not appear to alter the timing of centrosome separation, demonstrating that centriole disengagement has not been detectably perturbed. **F)** Images show the timing of centrosome separation in embryos expressing Jupiter-mCherry (*red)* and either wild type Sas-4-GFP or Sas-4-T200A-GFP (*green*), as indicated. Centrosome separation occurs with normal timing during telophase/early S-phase in embryos expressing Sas-4-T200A-GFP, confirming that the expression of this protein does not detectably perturb centriole disengagement, even though the loading of Polo-GFP onto new centrioles is strongly perturbed.

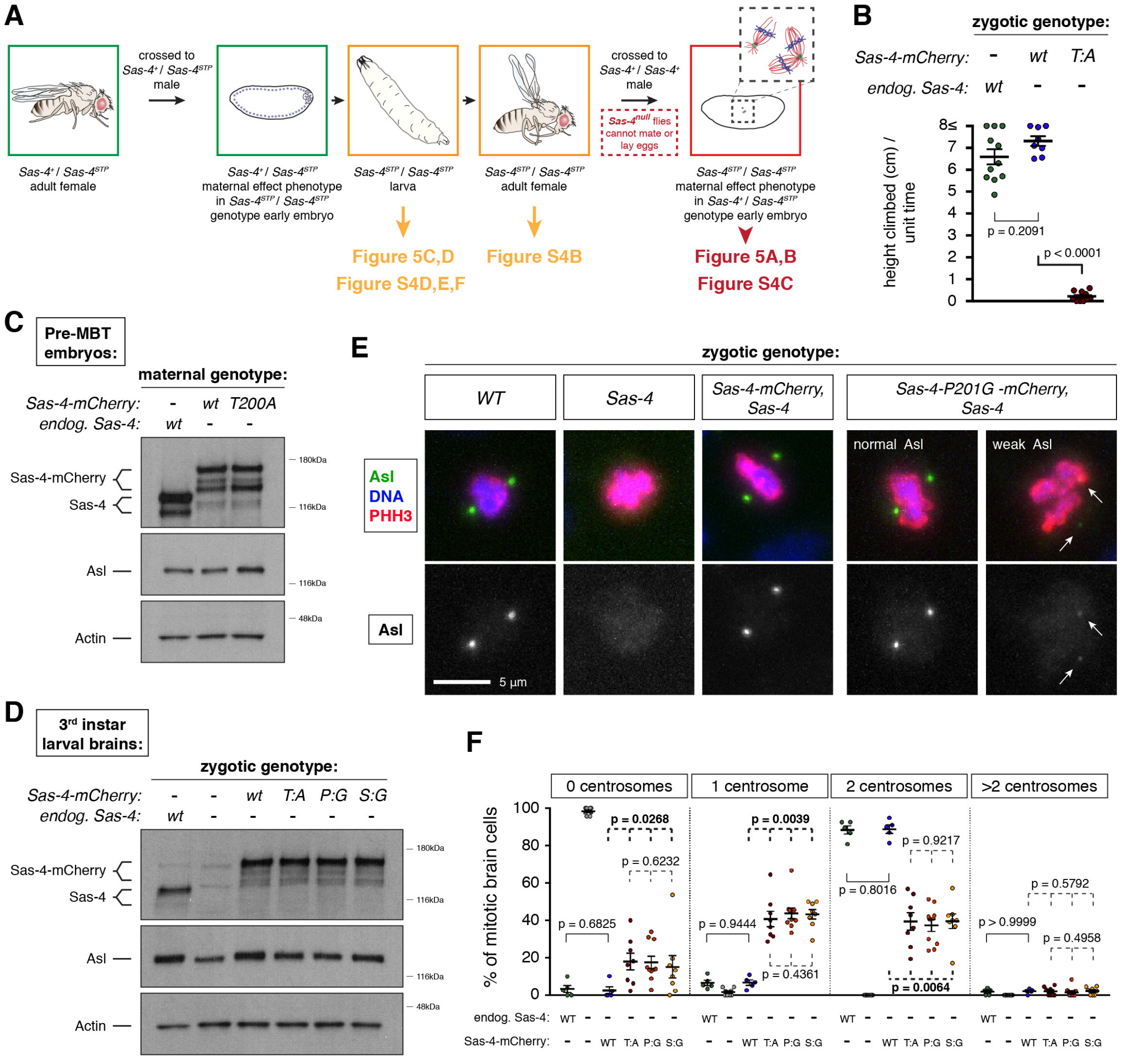

Figure S4, related to Figure 5.

Analysis of Sas-4-Thr200-STP-motif mutant transgenic flies. A) Schematics indicate the life cycle stages and the specific genotype-phenotype relationships in *Drosophila* that correspond to the different Sas-4-STP transgenic rescue experiments presented in this study. Green, orange and red boxes indicate increasingly severe phenotypical defects (green: minimal defects – red: lethality). **B)** Climbing assay of adult *Drosophila* measuring the proprioception of flies expressing transgenic Sas-4-mCherry or Sas-4-T200A-mCherry in a *Sas-4* null genetic background. *Sas-4* null flies are severely uncoordinated and completely unable to stand up therefore they were not subjected to the climbing assay. The graph shows that Sas-4-mCherry expression fully rescues this proprioception defect of *Sas-4* null flies (as measurable with this type of assay) while Sas-4-T200A-mCherry expression can only marginally rescue the *Sas-4* null phenotype. Error bars show SEM. Statistical analysis was performed using the Mann-Whitney test. **C)** Western blot shows that Sas-4-mCherry and Sas-4-T200A-mCherry are expressed at near-endogenous levels in pre-cellularised embryos and that levels of Asl are not detectably perturbed. Actin is shown as a loading control. Note that the multiple bands of Sas-4 visible on the blot are not phosphorylation-dependent (bands appear resistant to phosphatase treatment; data not shown). **D)** Western blot shows that in 3rd instar larval brains Sas-4-mCherry and the Sas-4-mCherry T200A, P210G and S199G mutant proteins are expressed at similar levels. The levels of Asl are also not detectably perturbed, although Asl levels are reproducibly slightly downregulated in *Sas-4* mutant brains that are not rescued by any of these transgenes. Actin is shown as a loading control. **E)** Images show examples of 3rd instar larval brain cells that were used for quantifying Asl foci in mitotic cells; genotypes are indicated. The centrosomal levels of Asl were often variable in flies expressing Sas-4-T200A-mCherry, Sas-4-P201G-mCherry or Sas-4-S199G-mCherry in the absence of wild type Sas-4 (an example of 2 cells from the same *Sas-4-P201G-mCherry,Sas-4* brain is shown—both cells have two centrosomes, but one has normal Asl levels, the other has low Asl levels, although both cells were scored as having two centrosomes). **F)** Scatter plot representation of graph shown in Figure 5D. Each data point represents an average value obtained from one brain (30-50 cells counted in each brain). Error

bars show SEM. Statistical analysis was performed using the Mann-Whitney test (pairwise comparisons; indicated with solid lines) or the Kruskal-Wallis test (comparison of 3 or 4 categories; indicated with dashed lines). Bold lines highlight significant differences between categories. Note that the variability in centrosome frequencies is far greater in flies expressing Sas-4-T200A-mCherry, Sas-4-P201G-mCherry or Sas-4-S199G-mCherry when compared to WT Sas-4-mCherry, suggesting that each of these mutations induces a stochastic failure in centriole conversion.

Supplemental Experimental Procedures

Generation of Sas-4 constructs for RNA microinjection

To generate the various Sas-4-GFP constructs used for RNA injection experiments the *Sas-4* coding region was PCR amplified from w^{67} DNA and subcloned into the pRNA-EGFP-CT (Cottee et al., 2015) vector using Gateway technology. The single intron of *Sas-4* was removed using QuickChange II XL Site-Directed Mutagenesis Kit and all described deletions or point mutations were subsequently introduced with QuickChange II XL or QuickChange Multi Site-Directed Mutagenesis Kits. To generate the Sas-4-mKate2 constructs the mKate2 coding sequence was PCR amplified from the pDONR P2R-P3-mKate2 vector (Buj et al., 2013) with flanking homology arms to the sequence surrounding the eGFP coding region within the pRNA-Sas-4-EGFP vector. An NheI restriction site was introduced into the pRNA-Sas-4-EGFP vector immediately upstream of the GFP start codon and In-Fusion cloning (Clontech) was used to swap the eGFP coding sequence for the mKate2 sequence using the NheI and SpeI restriction sites within pRNA-Sas-4-EGFP (the NheI site was not included in the homology arms when amplifying mKate2 and therefore was removed during the In-Fusion reaction to restore the original linker sequence). The T200A, P201G and S199G point mutations were introduced into pRNA-Sas-4-mKate2 as described in Experimental Procedures.

Phospho-antibody generation

The anti-Sas-4-pThr200 antibody was generated by Pocono Rabbit Farm & Laboratory Inc. A polyclonal antibody was raised in rabbit against a synthetic peptide with the sequence of SS{pThr}PDLKSSYASSC. Following affinity purification using the phospho-peptide, the antibody was purified against the non-phsophorylated peptide sequence SSTPDLKSSYASSC to eliminate any antibodies that are not specific to the phospho-epitope.

Antibody labelling and microinjection

The anti-Sas-4-pThr200 antibody was covalently coupled to Alexa488 or Alexa568 (succinimidyl ester forms, Life Technologies) by 1-hour incubation of the antibody and dye in 50mM HEPES pH 8.6, 50mMKCl, 1mM MgCl, 1mM EGTA. The labelled antibody was separated from unbound dye and eluted in 50mM HEPES pH 7.5, 50mMKCl, 1mM MgCl, 1mM EGTA using size exclusion chromatography and concentrated to approximately 5-10mg/ml using Amicon Ultra filters (50kDa MWCO). The antibodies were injected into syncytial blastoderm stage embryos and imaged immediately following injection using the spinning disc confocal system described in Experimental Procedures.

Image processing and presentation

All fluorescent images presented that were collected on the Perkin Elmer Spinning Disc confocal system (living embryos and brains) were obtained by 3D rendering of image data using Volocity software. The Volocity ‘smooth zoom’ filter was applied to all images. All immunofluorescence images of fixed embryos and brains were processed using Fiji software (Schindelin et al., 2012).

Peptides

For the kinase assays and Polo-Box binding assays, peptides (and, where appropriate, their phosphorylated equivalents) were synthesised by GenScript. The full peptide sequences were the following: biotin-Ahx-G-Ahx-GGAKNACS-{S/G}-{T/pT/A}-{P/G}-DLKSAKK (representing the *Drosophila* Sas-4-Thr200-STP-motif) and biotin-Ahx-G-Ahx-GGAGHRMSS-{T/A}-{P/G}-VKAVAKK (representing the human CPAP-Thr616-STP-motif). Ahx indicates aminohexanoic acid. Peptides were resuspended and stored in 0.1M phosphate buffer pH7.4, 150mM NaCl, 2mM DTT.

Antibodies used for Western blotting

Rabbit anti-Sas-4 (Basto et al., 2006), rabbit anti-Asl (Conduit et al., 2010), mouse anti-actin (Sigma) and rabbit anti-Sas-4-pThr200 (this study) primary antibodies were diluted 1:500 in milk solution for Western blot/dot blot experiments. HRP-conjugated anti-rabbit or anti-mouse (GE Healthcare; diluted 1:3,000 in milk solution) were used as secondary antibodies. Streptavidin-HRP (Thermo Scientific; diluted 1:3,000 in milk solution) was used for detecting biotin, as a loading control, for all *in vitro* experiments.

Adult *Drosophila* climbing assay

The climbing ability of wild type, *Sas-4-mCherry,Sas4*^{S2214}*/Df(3R)BSC221* and *Sas-4-T200A-mCherry,Sas4*^{S2214}*/Df(3R)BSC221* was analysed blind. Groups of five 1-day-old flies were place in a 15ml measuring cylinder and knocked to the bottom. After 6 seconds the distance climbed by each of the five flies was measured and averaged. Each group of flies was measured 3 times and a single data point was calculated as the average value of the three repeats. 8-11 groups of flies were scored for each genotype (total of 40-55 flies, 3 runs each), and groups were unblinded after quantification was completed. Statistical comparisons between the different genotypes were performed using the Mann-Whitney test.

Supplemental References

Buj, R., Iglesias, N., Planas, A.M., and Santalucía, T. (2013). A plasmid toolkit for cloning chimeric cDNAs encoding customized fusion proteins into any Gateway destination expression vector. BMC Mol. Biol. *14*, 18.

Conduit, P.T., Brunk, K., Dobbelaere, J., Dix, C.I., Lucas, E.P., and Raff, J.W. (2010). Centrioles regulate centrosome size by controlling the rate of Cnn incorporation into the PCM. *20*, 2178–2186.

Cottee, M.A., Muschalik, N., Johnson, S., Leveson, J., Raff, J.W., and Lea, S.M. (2015). The homo-oligomerisation of both Sas-6 and Ana2 is required for efficient centriole assembly in flies. Elife *4*, e07236.

Cottee, M.A., Muschalik, N., Wong, Y.L., Johnson, C.M., Johnson, S., Andreeva, A., Oegema, K., Lea, S.M., Raff, J.W., and van Breugel, M. (2013). Crystal structures of the CPAP/STIL complex reveal its role in centriole assembly and human microcephaly. Elife *2*, e01071.

Hatzopoulos, G.N., Erat, M.C., Cutts, E., Rogala, K.B., Slater, L.M., Stansfeld, P.J., and Vakonakis, I. (2013). Structural analysis of the G-box domain of the microcephaly protein CPAP suggests a role in centriole architecture. Structure *21*, 2069–2077.

Schindelin, J., Arganda-Carreras, I., Frise, E., Kaynig, V., Longair, M., Pietzsch, T., Preibisch, S., Rueden, C., Saalfeld, S., Schmid, B., et al. (2012). Fiji: an open-source platform for biological-image analysis. Nat Methods *9*, 676–682.
